# The Effects of Wildfire on Mortality and Resources for an Arboreal Marsupial: Resilience to Fire Events but Susceptibility to Fire Regime Change

**DOI:** 10.1371/journal.pone.0022952

**Published:** 2011-08-03

**Authors:** Sam C. Banks, Emma J. Knight, Lachlan McBurney, David Blair, David B. Lindenmayer

**Affiliations:** The Fenner School of Environment and Society, The Australian National University, Canberra, Australian Capital Territory, Australia; Institut Pluridisciplinaire Hubert Curien, France

## Abstract

**Background:**

Big environmental disturbances have big ecological effects, yet these are not always what we might expect. Understanding the proximate effects of major disturbances, such as severe wildfires, on individuals, populations and habitats will be essential for understanding how predicted future increases in the frequency of such disturbances will affect ecosystems. However, researchers rarely have access to data from immediately before and after such events. Here we report on the effects of a severe and extensive forest wildfire on mortality, reproductive output and availability of key shelter resources for an arboreal marsupial. We also investigated the behavioural response of individuals to changed shelter resource availability in the post-fire environment.

**Methodology/Principal Findings:**

We fitted proximity-logging radiotransmitters to mountain brushtail possums (*Trichosurus cunninghami*) before, during and after the 2009 wildfires in Victoria, Australia. Surprisingly, we detected no mortality associated with the fire, and despite a significant post-fire decrease in the proportion of females carrying pouch young in the burnt area, there was no short-term post-fire population decline. The major consequence of this fire for mountain brushtail possums was the loss of over 80% of hollow-bearing trees. The types of trees preferred as shelter sites (highly decayed dead standing trees) were those most likely to collapse after fire. Individuals adapted to resource decline by being more flexible in resource selection after the fire, but not by increased resource sharing.

**Conclusions/Significance:**

Despite short-term demographic resilience and behavioural adaptation following this fire, the major loss of decayed hollow trees suggests the increased frequency of stand-replacing wildfires predicted under climate change will pose major challenges for shelter resource availability for hollow-dependent fauna. Hollow-bearing trees are typically biological legacies of previous forest generations in post-fire regrowth forests but will cease to be recruited to future regrowth forests if the interval between severe fires becomes too rapid for hollow formation.

## Introduction

### Knowledge gaps on the proximate effects of disturbance on fauna

Natural disturbance events such as wildfires, tsunamis or volcanic eruptions can have widespread and long-term effects on ecosystems [1], [2], [3]. Natural disturbance regimes are predicted to change under future climate scenarios [4], with an increased frequency of severe wildfires in many terrestrial ecosystems [5], [6], [7], [8]. To predict the long-term biodiversity consequences of changed disturbance regimes, we need a process-based understanding of how species respond to disturbance events. Key knowledge gaps relate to the proximate effects of disturbances on demography, short and long-term resource availability and the adaptive responses of species to such environmental changes [9], [10]. These knowledge gaps are particularly relevant to high intensity disturbances such as severe wildfires, as the unpredictable nature of such events makes reliable studies (e.g. comparing affected and unaffected areas before and after disturbance) difficult to implement [11].

In this study, we quantified the effects of a major wildfire, the February 2009 Black Saturday wildfires in Victoria, Australia, on short-term survival, reproductive output and resource availability for the arboreal mountain brushtail possum (*Trichosurus cunninghami*). Further, we tested for behavioural adaptation of resource use patterns in the post-disturbance environment. The questions that are set out below and addressed in this paper were tractable because we were in the rare position of having detailed data on demography, individual resource use and resource availability from before and after an unexpected severe disturbance, and in disturbed and undisturbed habitat. Our study proceeded according to the following series of key questions:

### What is the short-term effect of wildfire on survival and reproductive output?

The distribution and abundance of survivors (*residuals* or *biological legacies*) is a critical factor influencing population recovery and post-disturbance community composition [4], [12]. For instance, the level of *in situ* survival after a disturbance event can determine the dependence of population recovery on immigration [13], [14]. In conjunction with the abundance of survivors, post-fire reproductive output is likely to be a key driver of population recovery. In the context of increasing fire frequency with climate change, high mortality associated with fire is likely to lead to declines in abundance and/or restriction to undisturbed refugia for species with low fecundity or recolonisation ability. Here, we quantified the effects of a major fire event on survival, abundance and reproductive output.

### Does wildfire affect shelter resource availability?

**Figure 1:**
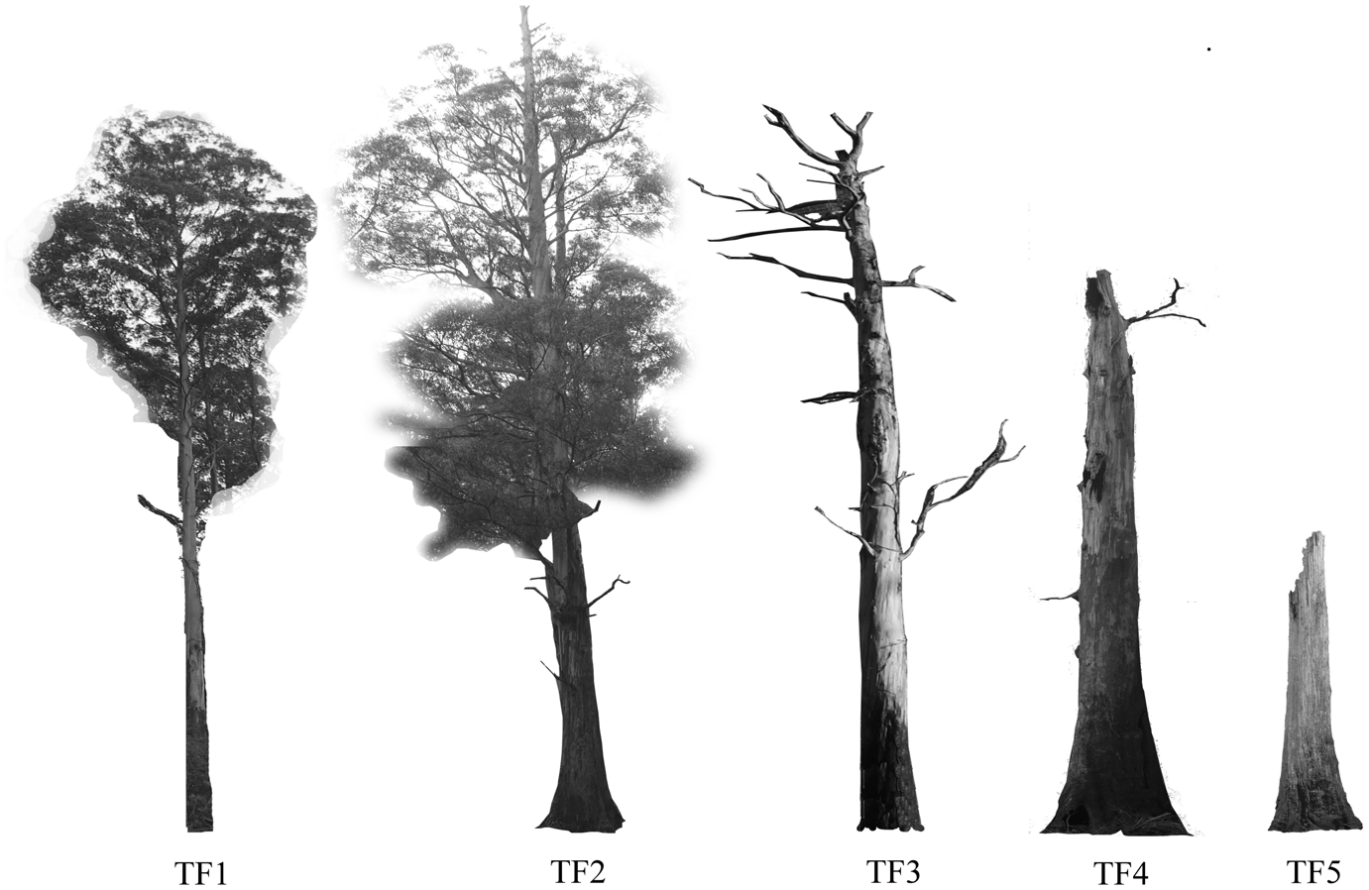
Stages of hollow formation, death and decay of mountain ash trees (*Eucalyptus regnans*). The trees surveyed in this study were characterised according to five tree form (TF) categories pictured from left to right: TF1: live trees with no visible hollows (typically young trees); TF2: live trees with visible hollows (typically older, senescing trees); TF3: dead trees in the early stages of decay; TF4; dead trees in the mid-stages of decay; and TF5: highly decayed dead trees. Live trees do not begin to form hollows suitable for arboreal marsupials until they reach an age of at least 120 years.

Disturbance has major effects on shelter resources for fauna [15]. Shelter availability has, in turn, a key influence on the survival and distribution of individuals in the post-disturbance environment [14], [16]. Many forest animals are dependent on tree hollows for shelter [17]. In Australian tall eucalypt forests, large hollows suitable for arboreal marsupials begin to form in live trees that are over 120 years old, and dead standing trees may provide useful hollows at all stages of decay until the complete collapse of the tree [18] (Figure 1). We focused on the effects of fire on hollow tree availability because hollow trees are a key resource whose availability limits the local abundance of several species, including the mountain brushtail possum [18]. Indeed, the decline of hollow-bearing trees due to activities such as logging is a key threatening process for arboreal marsupials [17]. The impacts of fire on tree hollow availability are undocumented in this system. We used pre and post-fire data to estimate the rate of loss of hollow bearing trees due to fire and to identify whether particular age classes (decay stages) of hollow bearing trees were susceptible to post-fire collapse. This enabled us to predict the long-term consequences of changed fire regimes for the availability of this key resource.

### Do animals show behavioural responses to post-disturbance environmental change?

Behavioural responses can mediate the impacts of disturbance on animal populations [19], [20]. Potential responses to post-fire resource decline include range-shifting to track the remaining resources [21]; reduced individual resource use; increased sharing of the remaining resources [22]; or flexibility in resource selection [20]. Behavioural responses to resource decline may mitigate or exacerbate the negative effects of disturbance. For instance, the documented social response of the mountain brushtail possum to the decline in availability of hollow trees was one of reduced cooperation and den sharing, which may constrain an adaptive resource-sharing response that would enable individuals to continue to access preferred resources under resource decline [22].

The questions that we addressed in this study enabled us to identify the proximate effects of severe disturbance on demography and resource availability, and to understand the strategies used by individuals to persist in post-disturbance landscapes. From these results, we were able to predict the long-term impacts of changed disturbance regimes on the availability of critical resources (hollow trees) for populations of arboreal fauna.

## Methods

### Ethics statement

The animal capture, handling and radiotracking procedures conducted during this research were approved by the Australian National University Animal Experimentation Ethics Committee (permit C.RE. 58.09).

### Study system

The ‘Black Saturday’ fires that began on 7 February 2009 in Victoria, Australia, affected over 3500 km^2^, a large proportion of which was high severity fire in tall eucalypt forest. While the fire was the most severe natural disaster in the State's recorded history, it fits within the disturbance regime of severe but infrequent stand-replacing fires in the forests of this region [23]. Such fire regimes occur in forest ecosystems worldwide, and are major drivers of forest structure and spatiotemporal variation in ecological communities [24], [25], [26], [27].

Our study focuses on the extensive tall forests dominated by mountain ash (*Eucalyptus regnans*), that occur between altitudes of 800 m and 1100 m in the Victorian central highlands. Mountain ash trees are the world's tallest angiosperms and are killed by severe fires that scorch or consume the forest canopy [28]. After approximately 100–150 years, mountain ash trees begin to develop hollows that are used as shelter by a variety of fauna, including several marsupial species [17]. The focus of this study is the mountain brushtail possum (*Trichosurus cunninghami*), a large (adults 2.5–4 kg), nocturnal and relatively long-lived (∼12 years) marsupial with a generalist diet [29].

**Figure 2:**
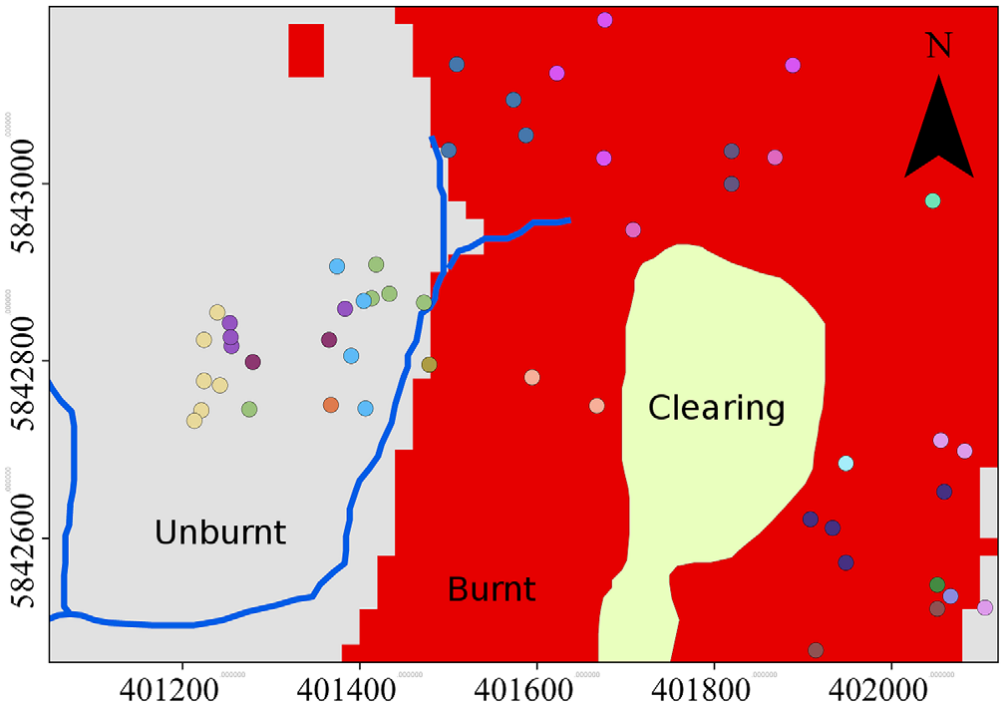
Study site map. The map shows the spatial extent of the Cambarville study site affected by fire in February 2009. The coloured dots represent hollow trees to which mountain brushtail possums (*Trichosurus cunninghami*) were radiotracked (different colours represent separate individuals). The X and Y axes are Eastings and Northings UTM coordinates in metres (Geodetic Datum of Australia). The pale yellow area in the centre of the site is a grassy clearing, the red area is the area burnt in the 2009 fire and the grey shaded area was not burnt.

We studied mountain brushtail possums over a ∼50 ha area at Cambarville (37°33.44 S, 145°53.05 E, 880–970 m altitude), the site of an ongoing long-term study of this species that began in 1992. Approximately half of the study area was burnt in February 2009 (Figure 2). The effect of the fire at this site was the full consumption of the understorey vegetation, consumption of leaves and smaller branches of the mid-storey, and some scorch of the crowns of the 50–85 m overstorey trees. Consumption by fire of the main stems of larger trees was dependent on pre-fire tree decay. This level of fire severity is similar to that across most of the surrounding landscape (at least within an approximate 5 km radius), although there were some small neighboring patches of forest burnt at much higher severity. The unburnt section of the site represents a small proportion (∼25%) of the local landscape (within a ∼5 km radius) that was not burnt. Due to topographic conditions, the area of the Cambarville site that was burnt in 2009 largely overlapped with the area that was burnt in a previous fire in 1939. Because the post-1939 regeneration trees had not yet formed hollows suitable for mountain brushtail possums, the 2009 burnt area had fewer hollow-bearing trees than the unburnt area, and those hollow-bearing trees that were present were dead and highly decayed legacies of the pre-1939 forest. In contrast, the forest that was unburnt in 2009 had a range of ages of hollow bearing trees, from live old growth trees to dead and decayed trees.

**Figure 3:**
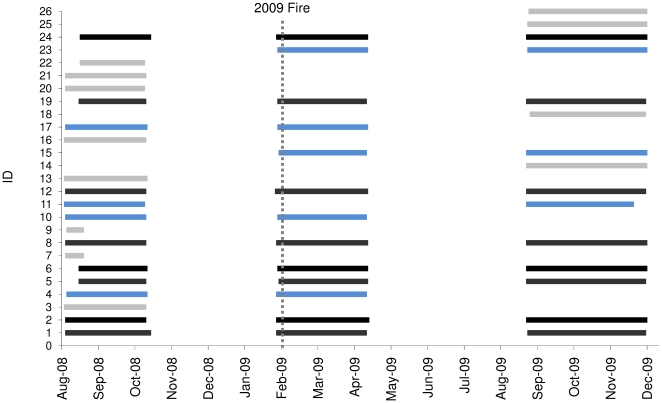
The duration over which each study animal was fitted with a proximity-logging radiotransmitter. Black bars represent individuals carrying transmitters for all three study periods, blue bars represent individuals carrying transmitters for two of the three periods and light grey bars represent individuals carrying transmitters for one study period. The vertical line shows the date that the Black Saturday wildfires commenced (7 February 2009). The collars fitted to animals in February 2009 were attached over the four days immediately preceding the fires. Individuals 1–6 were located in the unburnt section of the study site and individuals 7 to 26 were located in the burnt area of the study site (see Figure 2 for map).

We fitted proximity-logging radiotransmitters (Sirtrack Ltd, New Zealand) to 26 mountain brushtail possums over three periods before and after the February 2009 fire event: pre-fire (August to October 2008), pre-post-fire (February to April 2009) and post-fire (August to November 2009). The collars fitted to animals in February 2009 were attached over the four days immediately preceding the fires that began on 7 February 2009. The collars weighed 30 g, which is approximately 1% of the body mass of the study animals. Each collar contained a VHF transmitter (150–152 MHz) and UHF transmitter/data-logger to record proximity of other collared individuals. Not all individuals were fitted with collars in every study period, and Figure 3 shows the individuals fitted with collars in each period and the duration over which they were fitted. Over the study, 20 individuals were collared in the burnt area of the study site and six were collared in the unburnt area. The individuals fitted with collars were adults that were known to be resident at the site on the basis of extensive past trapping data [30], and all trapped individuals in this category were fitted with collars. The data we collected enabled us to answer the following questions:

### What was the short-term effect of wildfire on survival, abundance and reproductive output?

We estimated the rate of survival by the proportion of the 13 mountain brushtail possums to which we fitted radiotransmitters in early February 2009 (immediately before the wildfires of 7 February 2009) that were recaptured in April 2009. Eight of these individuals were in the area that was burnt and five were in the unburnt area (Figure 3). We limited this analysis to individuals that actually carried collars during the fire event, because the radiotransmitters were detectable over long distances, so the risk of incorrect inference of mortality due to emigration was relatively low (in any case, all were recaptured post-fire, so this issue did not arise). We also compared the overall number of individuals captured in the population before and after the fire from 15 trapping sessions conducted between March 2004 and November 2010. Each of these surveys comprised 55 traps set at consistent trapping locations across the Cambarville study site for a period of three nights [30]. Using this protocol, the recapture probability for individuals alive in this population has been estimated at approximately 80% [28].

We compared pre- and post-fire reproductive output of adult females in the burnt and unburnt areas with a logistic regression analysis of the probability of a female carrying a pouch young in August 2007–2010. Young are typically born around early April, and August is the time of year when the young are well-developed but still attached to the mother's teat (thus, reproductive status can be unequivocally identified for females). We used a generalised linear mixed model in Genstat 11 [31] to test for an interaction between *Post-fire* (August 2007 and 2008 = 0; August 2009 and 2010 = 1) and *Burnt_zone* (Unburnt area  = 0, burnt area  = 1) on the probability of carrying a pouch young. We fitted individual ID and sampling occasion (year) as random effects. Through initial exploratory analyses, we found that female age had no effect once we excluded juveniles, so this was not incorporated in the models presented here.

### Did fire affect the availability of hollow bearing trees?

Before the fire, we characterised 110 trees according to five ‘tree forms’ relating to the stage of tree decay and hollow formation (TF1–5 in Figure 1). Half of these trees were known from radiotracking records to be used as dens by possums and were typically older trees (TF2–5, Figure 1) and half of the trees were randomly selected within approximately 20 m of each characterised den tree. These were typically younger trees (TF1–5, Figure 1). Whether or not a tree was used as a den had no bearing on the degree to which it was damaged by fire, and this was not a factor in the analysis. However, this sampling scheme resulted in adequate representation of all tree forms (particularly those used by mountain brushtail possums) in the analysis. After the fire, we characterised fire damage to these trees as scorch (no obvious fire consumption of the main stem or branches), partial consumption (tree was still standing and potentially had some utility as a den site), or complete consumption/collapse (tree had lost all utility as a den site).

The analysis of fire damage to trees was restricted to the 56 trees characterised in the burnt section of the study area, as no trees were lost in the unburnt area. Thirty five of these 56 trees were known to function as den trees. We condensed tree forms TF3–5 (Figure 1) into a single category (dead trees). We used the *polr* function in the MASS package [32] of R 2.12 [33] to fit an ordinal logistic regression model of the relationship between fire damage (the response variable) and pre-fire tree form. We did not consider other variables such as fire severity in this analysis, as the fire within the burnt section of this 50 ha study site was of a relatively uniform severity in relation to the variation in fire severity over the approximate 3500 km^2^ affected by the fire. We simply classed trees as being in burnt or unburnt habitat.

### Was there a behavioural response to the modified post-disturbance environment?

#### Did individuals shift in denning range after fire?

We tested for a spatial shift by individuals in the area used for denning before and after the fire with a randomisation test that compared the distance between the centroids (mean coordinates) of the geographic coordinates of each record of den tree use before (Aug–Oct 2008) and after the fire (Aug–Nov 2009) in burnt and unburnt habitat. The test featured 1000 randomisations of the timing of the pre/post-fire breakpoint while keeping the temporal order of den use records static. This analysis was restricted to the five individuals in burnt habitat and four individuals in unburnt habitat that were fitted with radiotransmitters in both of these periods.

#### Did individuals use the same number of den trees before and after fire?

We fitted linear mixed models in Genstat v11 [31] to investigate the number of different den trees used by the nine individual possums (five in burnt habitat and four in unburnt habitat) that were fitted with radiotransmitters in the winter-spring before the fire (August–October 2008) and the winter-spring after the fire (August-November 2009). Individual ID of the study animals was included as a random term in the model and we tested for an interaction between *Burnt_zone*, a binary spatial variable distinguishing the area that was burnt (1) from the area that was not burnt (0), and *Post_fire*, a binary temporal variable distinguishing the pre and post-fire periods. We also tested for potential effects of sex and the number of radiotracking fixes obtained per individual.

#### Did the rate of den sharing change after fire?

Mountain brushtail possums shelter in tree hollows during daylight hours and do not change dens during the day [22], [34]. We used the proximity-logger collars to quantify the rate of day-time den-sharing among individuals in August-October 2008 (pre-fire: 20 individuals) and August-November 2009 (post-fire: 15 individuals). Full details of the preparation of interaction data from the UHF proximity-loggers are presented elsewhere [22]. We used generalised linear mixed models in Genstat 11 [31] with logit link function to test whether distance (between the geographic centroids of each individual's radiotracking locations) and an interaction between *Burnt_zone* (spatial) and *Post_fire* (temporal) influenced the probability of diurnal den sharing between each pair of individuals. The binary response variable represented instances of den-sharing (1) or non-sharing (0) for each pair of individuals on each day in which both individuals were collared. For the logistic regression, the binary response was coded as the number of den sharing days over the total possible number of den-sharing or non-sharing days. Only pairs in which both individuals were in either the burnt or unburnt zone were included in the analysis. Exploratory analyses that incorporated temporal correlation between observations on subsequent days yielded similar results to those presented here, and we found little support for the inclusion of such correlation structures in our models. We acknowledge that our model does not account for potential non-independence of den-sharing probabilities between different pairs that have one individual in common. This may occur if the den being used is too small to accommodate a third individual, or if the social relationship between one pair influences the chance of sharing in the other pair (e.g. one individual ‘brings a friend’ to share with another). However, given that the overall den-sharing rate that we documented was so low (see Results), and that individuals tended to share with only one other individual throughout the study, the chance of ‘interference’ between multiple potential den-sharers was minor.

#### Did shelter resource selection change from before to after the fire?

We used records of the tree form selected by the nine individuals (five in burnt habitat and four in unburnt habitat) that were fitted with collars both in the winter-spring before the fire (August-October 2008) and the winter-spring after the fire (August-November 2009) to code a set of response variables that enabled us to test for a shift in tree form selection by possums in the burnt zone after the fire. The three response variables were binary, and represented cut points in the tree form progression from TF2 to TF3–5, TF2–3 to TF4–5, and TF2–4 to TF5, respectively. TF1 was not included in this analysis as trees in this category did not have hollows and were not used as dens (except on very few occasions). We used logistic regression models with individual ID as a random term in Genstat 11 [31] to test the effect of a spatiotemporal interaction between *Burnt_zone* and *Post_fire*.

## Results

### What was the short-term effect of wildfire on survival, abundance and reproductive output?

**Figure 4:**
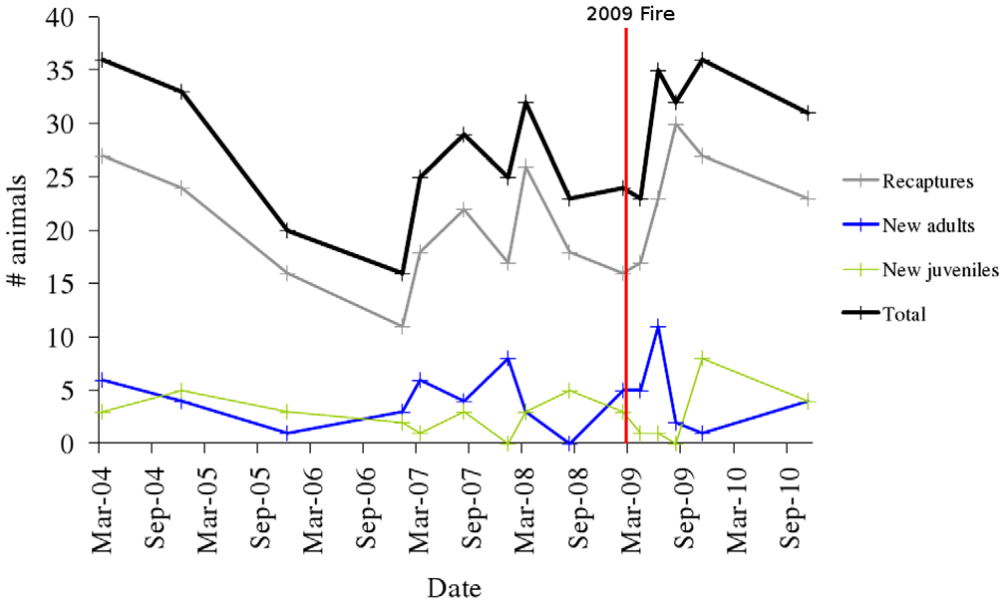
Demographic trends. The number of mountain brushtail possums captured at the Cambarville study site before and after the February 2009 Black Saturday wildfires. The timing of the15 trapping sessions over this period are indicated with a “+” sign.

All 13 individuals fitted with radiotransmitters immediately before the wildfire (February 2009) were recaptured alive in April 2009 (Figure 3). Those same 13 individuals were also recaptured alive in August 2009 and tracked throughout late 2009. The abundance of mountain brushtail possums across the entire study site remained approximately stable between the trapping sessions immediately before and after the fire (24 and 23 animals, respectively), then increased in mid 2009 and remained stable at the higher levels in subsequent trapping sessions (Figure 4). The mean number of animals captured on seven trapping sessions from Jan 2007 to Feb 2009 was 24.4 (±5.3 s.d.), increasing to 33.5 (±2.4 s.d.) over four trapping sessions from June 2009 to Dec 2010 (T-test, P = 0.004). This increase occurred predominantly in the unburnt habitat, where the mean number of animals captured per session increased 71.1% from 6.43 (±1.39 s.d.) over seven trapping sessions from Jan 2007 to Feb 2009 to 11.0 (±3.81 s.d.) over five trapping sessions from April 2009 to Dec 2010 (T-test, P = 0.005). In the unburnt area, the mean ratio of new (previously uncaptured) adults to recaptures changed from 0.29 (±0.48 s.d.) to 0.46 (±0.64 s.d.) per session from before to after the fire, whereas the ratio of new juveniles to recaptures changed from 0.18 (±0.23 s.d.) to 0.12 (±0.22 s.d.). In the burnt area, the increase in mean capture numbers per session was 15% over the same period, from 16.0 (±4.73 s.d.) to 18.4 (±1.82 s.d.) (T-test, P = 0.098). In the burnt area, the mean ratio of new (previously uncaptured) adults to recaptures changed from 0.32 (±0.23 s.d.) to 0.21 (±0.17 s.d.) per session from before to after the fire, whereas the ratio of new juveniles to recaptures changed from 0.14 (±0.14 s.d.) to 0.13 (±0.11 s.d.).

The proportion of captured females carrying pouch young in August 2007–2010 was 0.92 (n = 13) in 2007, 0.75 in 2008 (n = 8), 0.84 in 2009 (n = 19) and 1.00 in 2010 (n = 6). A generalised linear mixed model of the reproductive status of females identified a significant negative interaction between *Post_fire* and *Burnt_zone* (Interaction: F = 10.39, d.f. = 49, P = 0.002; Main effects: *Post_fire*: F = 0.67, d.f. = 49, P = 0.416; *Burnt_zone*: F = 0.24, d.f. = 49, P = 0.627). This model reflected a decrease in the percentage of females carrying young in the burnt habitat (from 100% pre-fire to 62.5% post-fire) and, unexpectedly, an increase in the unburnt habitat (from 57.1% to 100%). Thus, although the number of individuals captured increased in the burnt and unburnt areas after the fire, the relatively greater apparent demographic increase in the unburnt area was predominantly due to an influx of new adults and an increase in reproductive output by females in the unburnt habitat.

### Did fire affect the availability of hollow bearing trees?

**Figure 5:**
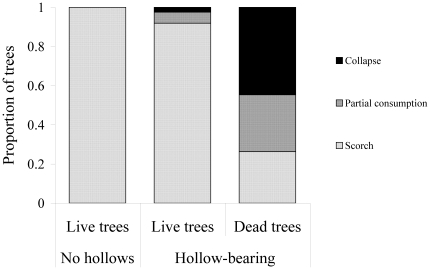
Effects of fire on mountain ash trees at different stages of hollow formation and decay. The chart shows the probability of a tree being scorched (no structural damage), partly consumed (tree is still standing and potentially has some utility as a den site), or completely consumed (tree has collapsed or has lost all utility as a den site) as predicted by ordinal regression.

The ordinal logistic regression model revealed a significant increase in the probability of partial or complete consumption of a tree with a change in pre-fire tree form to the later stages of decay (likelihood ratio test: P = 8.4×10^−9^). Trees that were alive before the fire suffered little fire damage, while most dead trees collapsed or were seriously consumed by the fire (Figure 5). Of the trees that we observed to be used by the mountain brushtail possum before the fire, only 7% of those in the burnt area were considered suitable as den trees after the fire and all dead trees in the late stages of decay (TF5) had collapsed.

### Was there a behavioural response to the modified post-disturbance environment?

#### Did individuals shift in denning range after fire?

We found a significant shift in the denning range centroids of individuals in the burnt zone from before to after the fire (Mean distance  = 138 m, P = 0.002). These post-fire shifts always resulted in a high degree of spatial overlap in den use with the pre-fire denning range, or a contraction to a sub-section of the pre-fire den-use range. To put the scale of pre to post-fire ‘movement’ in context, previous findings from this study site found the mean den-use range area of mountain brushtail possums to be 2.6 ha [35]. No such pre- and post-fire shift was detected among individuals in the unburnt zone (P = 0.388).

#### Did individuals use the same number of den trees before and after fire?

There was no evidence for an effect of fire on the number of den trees used per individual (*Burnt_zone* * *Post_fire* interaction: Wald statistic  = 0.05, d.f. = 1, P = 0.815). Also, there was no significant change in the number of trees used by individuals across the site (in the burnt and unburnt areas) before or after the fire (*Post_fire*: Wald statistic  = 0.026, d.f. = 1, P = 0.609). However, a consistent pattern throughout the duration of the study was that individuals in the area that was burnt used fewer den trees (before and after the fire) than those in the area that was not burnt (*Burnt_zone*: Wald statistic  = 12.1, d.f. = 1, P<0.001). This model predicted that individuals in the unburnt area used 3.825 (±0.362 s.e.) dens per study period (pre- or post-fire) and individuals in the burnt area used 2.360 (±0.248 s.e.) dens per study period. This spatial difference in den use most likely reflects a difference in the pre-fire availability of dens in each of the two areas, which was significantly lower in the area that was subsequently burnt (T test: P = 0.0013). Before the 2009 fire, the mean number of hollow-bearing trees within a 1 ha circle around the centroid of each possum's radiotracking-based locations was 17.02 (±6.81 s.d.) in the unburnt area and 5.71 (±1.70 s.d.) in the area that was burnt. This is because the 2009 unburnt area was ‘topographically sheltered’ and was less affected by the previous (1939) major wildfire in the region, and thus contained more old trees with hollows (TF2 in Figure 1). Neither gender nor the number of radiotracking fixes per animal influenced the detected number of dens used by an individual within a study period (although the latter varied little between individuals).

#### Did the rate of den sharing change after fire?

The overall probability of den-sharing between pairs of individuals was 0.016 (490 sharing records from 31452 pairs x nights), and individuals tended to share dens with only a single other individual over the course of the study (mean  = 1.41, max. = 4). Only nine individuals were found to share a den with more than one other individual (always on separate nights), and the instances of sharing with these ‘non-preferred’ individuals comprised only 16% of the recorded instances of den-sharing. We found no significant difference in the den sharing rate before and after the fire (*Post_fire:* Wald statistic  = 1.75, d.f. = 1, P = 0.189) and no significant interaction between *Burnt_zone* and *Post_fire* (Wald statistic  = 1.09, d.f. = 1, P = 0.299). However, throughout the duration of the study, we found a (marginally) significantly lower rate of den sharing in the area that was burnt than in the area that was not burnt (Wald statistic  = 4.30, d.f. = 1, P = 0.042). For individuals whose denning range centroids were 40 m apart (an arbitrary distance used for generating model predictions), the predicted probability of den sharing in the burnt zone (0.08±0.02 s.e.) was approximately one third of the probability of den sharing between equally proximal individuals in the unburnt zone (0.22±0.04 s.e.) before and after the fire. Proximity (between the centroids of radiotracking locations of each individual) had a significant effect on den sharing probability (Wald statistic  = 38.92, d.f. = 1, P<0.001). The coefficient for log transformed distance of −4.066 (±0.635 s.e.) equates to a 58-fold decrease in the odds of den-sharing for every 10-fold increase in the distance between the centroids of those individuals' home ranges.

#### Did shelter resource selection change from before to after the fire?

**Figure 6:**
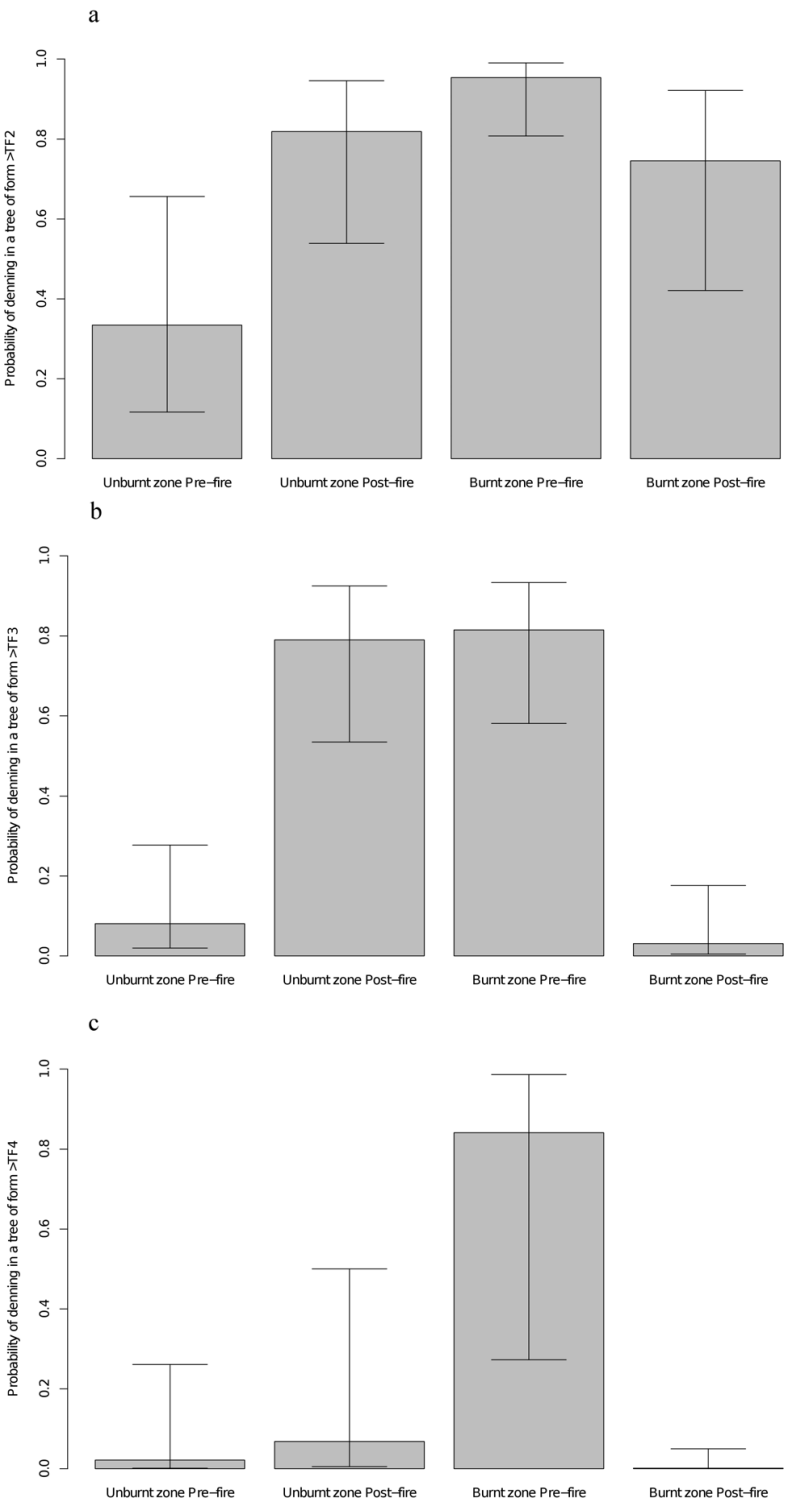
Pre and post-fire shifts in the tree forms used as shelter by mountain brushtail possums. These graphs show the predicted probabilities (and 95% confidence intervals) of an occupied tree being of a form greater than Tree Form 2 (Figure 6a), greater than Tree Form 3 (Figure 6b) or greater than Tree Form 4 (Figure 6c) before and after the fire unburnt and unburnt habitat. See Figure 1 for details of Tree Form (TF) categories. These predictions were from generalised linear mixed models of the types of trees used by individuals radiotracked before and after the fires. See Table 1 for fitted model statistics.

We found a significant post-fire shift to the use of younger, less decayed tree forms as dens by the individuals in burnt habitat. Most strikingly, the probability of an occupied den tree in the burnt area being a highly decayed tree (TF5 in Figure 1) decreased from 0.84 (95% CI 0.27–0.97) before the fire to 0.0015 (95% CI 0.00005–0.05) after the fire (Figure 6). This ‘downward shift’ in tree form selection in post-fire burnt habitat was significant, irrespective of where we placed the cut-point in tree form categories for the logistic regression (significance tests in Table 1, model predictions in Figure 6). After the fire, we observed possums in the burnt habitat to occasionally use tree forms not previously selected as dens, including three records of exposed ‘perches’ on the tops of narrow diameter (∼40 cm) broken tree trunks.

**Table 1:** Logistic mixed models testing for upward shifts in the tree forms (TF2–5, see Figure 1 for details) selected as den sites by mountain brushtail possums at Cambarville before and after the February 2009 Black Saturday fires.

Test	Fixed term	Wald statistic	d.f.	P	Estimate	SE
TF2 *vs* TF3–5	*Intercept*				0.985	0.489
	*Burnt_zone*	2.55	1	0.110	1.463	0.881
	*Post_fire*	5.36	1	0.021	0.787	0.413
	Burnt_zone * Post_fire	20.85	1	<0.001	−4.114	0.901
TF2–3 *vs* TF4–5	*Intercept*				−0.739	0.439
	*Burnt_zone*	0.19	1	0.662	−0.427	0.849
	*Post_fire*	2.65	1	0.103	0.035	0.535
	Burnt_zone * Post_fire	63.44	1	<0.001	−8.881	1.115
TF2–4 *vs* TF5	*Intercept*				−2.610	0.933
	*Burnt_zone*	3.09	1	0.079	0.585	1.503
	*Post_fire*	0.46	1	0.497	−2.092	0.824
	Burnt_zone * Post_fire	20.08	1	<0.001	−8.010	1.787

The significant negative interactions between *Burnt_zone* and *Post_fire* indicates that individuals in the burnt area used less-decayed trees after the fire.

We also found that possums in the unburnt zone were unlikely to use dead trees (TF3–5) as dens before the fire (probability  = 0.08, 95% CI 0.02–0.28), but used these kinds of trees often after the fire (probability  = 0.79, 95% CI 0.53–0.92). This was unexpected as there was no change in the availability of hollow bearing trees of any form in the unburnt habitat over the course of the study.

## Discussion

### Unexpected proximate effects of wildfire

An understanding of the proximate effects of disturbance events on natural populations is critical for predicting the ecological impacts of future changes in fire regimes [9], [10]. The short-term effects that we observed following the widespread February 2009 Black Saturday wildfires in south-eastern Australia were highly unexpected. Despite the extraordinary severity of this fire, we identified no mortality of mountain brushtail possums, nor short-term demographic decline. Indeed, there was an apparent increase in mountain brushtail possum abundance, predominantly in the unburnt area. This could either be due to preferential settling of juvenile dispersers in unburnt habitat, or to post-fire ‘refugee’ adults moving from burnt territories. The capture data suggest the occurrence of the latter immediately after the fire (Figure 4: June 2009) and the former slightly later (Figure 4: November 2009). While none of our collared individuals moved from the burnt to the unburnt area post-fire, these individuals did show significant (but not large) range shifts, suggesting that post-fire shifts from burnt to unburnt habitat may have occurred and could partly explain our demographic findings.

The primary effect of fire was not on the animals themselves, but on the availability of a critical resource, hollow-bearing trees. In the short-term, the population appeared resilient to the loss of this resource. This was due to behavioural flexibility in den resource selection, but not to an increase in den-sharing, despite the loss of over 80% of the hollow-bearing trees in the burnt habitat. Resource sharing following resource loss is most likely constrained by the increasing territoriality and reduced tolerance (particularly of non-kin) that occurs in response to shelter resource limitation in this species [22].

### Short-term consequences – demography, resource availability and behavioural adaptation

The abundance and distribution of survivors is a key factor influencing population recovery and spatiotemporal community dynamics [13], [14], [36]. Our results suggest that single fire events do not lead to major spatiotemporal variation in the abundance of mountain brushtail possums, as the post-fire population did not decline and predominantly consisted of *in situ* survivors (Figure 4). The high survival rate contrasts with that of other mammals following the Black Saturday wildfires. For instance, populations of the bush rat (*Rattus fuscipes*) were reduced by approximately 90% in burnt compared to unburnt habitat [14], and recent surveys suggest that recolonisation from unburnt habitat is a major contributor to demographic recovery for some small mammals, leading to spatial gradients in post-fire abundance (Banks, Blair, McBurney and Lindenmayer, unpublished data). Survival after disturbance events is likely to be strongly linked to life history attributes such as mobility, shelter use and dietary flexibility [37]. For instance, a flexible generalist diet is likely to confer increased survival after wildfires [38], while dietary specialists may be susceptible to the loss of food resources. The mountain brushtail possum has a very broad generalist diet [29], and the 100% survival of radiotracked individuals through the period immediately after the fire (when food availability is likely to be lowest) suggests that limitation of food resources is not a key factor influencing the post-fire demography of this species. In contrast, the greater glider (*Petauroides volans*) has largely disappeared from areas burnt by the 2009 conflagration, and this is likely to be at least partly attributable to this species' specialist dietary requirement of eucalypt leaves, which were lost due to scorch or consumption by fire [39].

It is possible that, despite high short-term survival, fire may have medium-term effects on the demography of mountain brushtail possums. While there was a major increase in apparent abundance after the fire, this was predominantly in the unburnt area, which received an unusually high number of adult recruits. These are potentially ‘refugees’ from adjacent severely burnt habitat, as the unburnt section of the Cambarville site was a relatively small green patch in a large burnt landscape. The reproductive output of females in the burnt habitat decreased in the two years following the fire (in contrast to the increase in the unburnt habitat). Individuals may live for at least 13 years, and locally-born young are recruited into the trappable population at approximately one year of age, while immigrants tend to be at least two years old. If the reduced reproductive output of females in the burnt area continues, a demographic decline may become evident in this area over the longer term. Any further conclusions about reproductive output and abundance would require further study, as it is possible that the reproductive output of females will recover to pre-fire levels in the relatively early stages of forest recovery.

The key proximate effect of fire that we identified over the timescale of our study was the loss of over 80% of the shelter resource in the burnt habitat. The extent to which trees were affected by fire was dependent on pre-fire decay stage, with live trees typically receiving only minor scorch with no ‘structural’ damage and dead trees typically collapsing or being consumed to a large degree. This has important implications for the degree of loss of hollow-bearing tree in forest stands with different fire histories. The burnt area that we studied lost most of its hollow-bearing trees because those that were present before the 2009 fire were highly-decayed dead trees that were killed in a previous fire in 1939. The post-1939 regrowth live trees had not yet formed hollows. In contrast, if the unburnt area of the Cambarville site had been burnt by the 2009 fire, we would expect a greater proportion of the hollow-bearing trees to have remained standing, since this area was not burnt in 1939 and many of the hollow-bearing trees were still alive or in the earlier stages of decay. Thus, the fire history of a forest stand influences the effect of individual fire events on hollow availability.

Because of the decay-dependent effect of fire on trees, and the preference of mountain brushtail possums on late-stage decayed trees [40], 93% of the trees known to be used by possums before the fire were lost from the burnt area post-fire. Despite this major resource loss, individuals in this area showed a range of behavioural responses that resulted in the maintenance of the number of dens used and the den-sharing rate from before to after the fire, presumably enabling the survival of these individuals despite the major loss of hollow-bearing trees. The key behavioural response was a shift, or relaxation, in resource selection within broadly the same spatial range of den use that those individuals used before the fire. Resource selection flexibility has been observed to confer demographic resilience to disturbance in other species [20]. However, we did not expect a shift in resource selection to be the ‘preferred’ response to a reduction in den availability, as the use of suboptimal den sites presumably confers reduced thermoregulatory benefits and increased predation risk (e.g. from owls). We expected that increased sharing of the remaining high quality den trees would be the ‘optimal’ response, but this was not observed. Individual fitness comes before the greater (population) good when making behavioural decisions [41], and our findings are consistent with theoretical, simulation and laboratory studies that show that cooperation with, and tolerance to others decreases as resource competition increases [42], [43]. Before the fire, the response to pre-fire spatial variation in den availability was that individuals in areas of relatively low local den availability (i.e. relatively high competition for dens) shared dens less often, were less tolerant of den-sharing with non-kin and more tolerant of kin (*i.e.* kin selection) [22]. Thus, the behavioural response to resource loss following fire involved the maintenance of social patterns (lack of cooperation in resource-poor habitat) at the expense of optimal resource selection (sharing of high-quality den trees).

Interestingly, there was also a shift in den tree selection in the unburnt area towards more highly decayed trees. Potential explanations for this include (1) changes in resource selection associated with increased resource competition due to the demographic increase in this area, and (2) changes in the thermal requirements of animals tracked under different temperature regimes. Hollows in trees of different decay classes are likely to have different insulative properties, and the mean minimum temperature on days that we radiotracked in Aug-Oct 2008 was 4.8°C (±4.2 s.d.) compared to 10.6°C (±4.5 s.d.) in Aug-Nov 2009.

### Long-term predictions – fire regime change, hollow trees and arboreal fauna

Our finding of no demographic decline associated with major hollow tree loss after wildfire contrasts with previous research indicating that the local availability of hollow-bearing trees has a significant positive effect on the abundance of mountain brushtail possums [44]. The surveys for the previous work did not take place immediately after a major reduction in tree hollow availability. Therefore, one possible explanation for this discrepancy between the studies is that tree hollow availability limits mountain brushtail possum abundance, but there is a temporal lag in the demographic decline after major reductions in hollow availability, such as that seen after the 2009 fire. If the latter is correct, the intregration of our findings on post-fire hollow tree loss with climate change-based predictions of increased future fire frequency [5], [6] suggests that hollow tree loss will be a major conservation issue for this species in future.

Under the current fire regime, although mountain ash trees are killed by severe fires [28], hollow-bearing trees are maintained in the landscape because stand-replacing fires occur at return intervals greater than the time required for live trees to form hollows (120–150 years). The most recent large fires in this region occurred in 2009, 1983 and 1939. However, these were not stand-replacing fires across the entire landscape. A proportion of the landscape was not burnt at all by any of these fires and some areas were burnt at low severity, such that the overstorey trees survived. Under this regime, many parts of the landscape that are burnt leave a biological legacy of numerous dead standing trees that will form additional hollows as they decay and thus function as critical shelter resources for fauna in young regrowth forests [45]. Thus, the immediate loss of highly-decayed dead trees after fire is likely to cause only a relatively short-term bottleneck in hollow tree availability.

Climate-based predictions of future fire regimes suggest that the frequency and severity of fires in this region and other forest environments will increase in future [5], [6], [7]. If the frequency of stand-replacing wildfires increases to the extent that mountain ash trees that germinated after a previous fire have not yet begun to form hollows before being burnt, there will be no biological legacies [12] in the form of large diameter dead trees that will function as shelter resources for fauna in regrowing forest stands. Further, there will be a rapid loss of the existing hollow-bearing trees. This is because the only hollow-bearing structures that young, regenerating forests currently contain are highly-decayed biological legacies of older forests that existed before the previous fire event (typically TF 4–5: Figure 1). As our results show (Figure 5), these have little chance of persisting through a subsequent fire. Thus, shortening return intervals of severe fires will remove dead hollow trees from young forest stands and prevent the post-fire recruitment of hollow bearing trees by reducing the age of stands that are burnt.

In tall forest ecosystems, the increased fire frequency associated with climate change may be exacerbated by a positive feedback loop between fire and forest age. Fire creates young regrowth forest by killing mature overstorey trees, and there is strong evidence from many forest ecosystems that young forests are more fire prone and burn at higher intensity than old forests [46], [47]. This future scenario of the loss of hollow trees due to changed fire regimes is particularly important in synchrony with the current clearfell logging regime. Clearfell logging directly removes hollow-bearing trees, prevents their recruitment (through rapid logging intervals) and increases the risk of fire in the landscape through changed forest structure (increased density of stems and crowns) and fuel loads (collapsed stems and branches during the early regrowth phase) [48]. Further, areas that are ‘topographically sheltered’ from fire are commonly subjected to logging, and the combination of these two disturbance types has removed much of the heterogeneity in forest stand age from this landscape. Indeed, old growth mountain ash forest now covers an estimated 1.13% of the Victorian central highlands region (Department of Sustainability and Environment, Victoria, Australia), whereas it was once likely to have been the most common type of forest in this landscape. Thus the synchronous effects of clearfell logging and wildfire are leading to rapid declines in the availability of hollow bearing trees. Ongoing monitoring to identify the longer-term demographic effects of the 2009 fire event will provide important data for understanding the likely outcomes of such landscape-scale drastic declines in hollow availability for arboreal marsupials.

### Conclusions

Understanding the proximate effects of disturbance on animal populations is essential for predicting how changed disturbance regimes will affect biodiversity. The proximate effects of wildfire on mountain brushtail possums were not on survival, but on resource availability. In the short-term, behavioural adaptation to reduced resource availability (flexibility in resource selection but not post-disturbance dispersal or increased resource sharing) enabled individuals to persist in the post-fire environment. The susceptibility of decayed dead trees to collapse after fire led to predictions of rapid decline of this key resource if fire return intervals shorten below that required for the recruitment of new hollow bearing trees. This is likely to be a major threat to the persistence of hollow-dependent fauna under future climate and disturbance scenarios.
